# Synthesis and Biological Evaluation of Amphotericin B Formulations Based on Organic Salts and Ionic Liquids against *Leishmania infantum*

**DOI:** 10.3390/antibiotics11121841

**Published:** 2022-12-19

**Authors:** Ricardo Ferraz, Nuno Santarém, Andreia F. M. Santos, Manuel L. Jacinto, Anabela Cordeiro-da-Silva, Cristina Prudêncio, João Paulo Noronha, Luis C. Branco, Željko Petrovski

**Affiliations:** 1 Chemical and Biomolecular Sciences, School of Health, Polytechnic Institute of Porto, 4200-072 Porto, Portugal; 2 LAQV-REQUIMTE, Department of Chemistry and Biochemistry, Faculty of Sciences, University of Porto, 4169-007 Porto, Portugal; 3 Institute for Research and Innovation in Health (i3S), University of Porto, 4200-135 Porto, Portugal; 4 LAQV-REQUIMTE, Department of Chemistry, NOVA School of Science and Technology, NOVA University of Lisbon, 2829-516 Caparica, Portugal; 5 Laboratory of Microbiology, Department of Biological Sciences, Faculty of Pharmacy, University of Porto, 4050-313 Porto, Portugal; 6 Center for Translational Health and Medical Biotechnology Research, Polytechnic Institute of Porto, 4200-465 Porto, Portugal

**Keywords:** organic salts and ionic liquids, amphotericin B, active pharmaceutical ingredients, *Leishmania infantum*, leishmaniasis

## Abstract

Nowadays, organic salts and ionic liquids (OSILs) containing active pharmaceutical ingredients (APIs) are being explored as drug delivery systems in modern therapies (OSILs-API). In that sense, this work is focused on the development of novel OSILs-API based on amphotericin B through an innovative procedure and the evaluation of the respective biological activity against *Leishmania infantum*. Several ammonium, methylimidazolium, pyridinium and phosphonium organic cations combined with amphotericin B as anion were synthesized in moderate to high yields and high purities by the water-reduced buffer neutralization method. All prepared compounds were characterized to confirm the desired chemical structure and the specific optical rotation ([α]*_D_*^25^) was also determined. The biological assays performed on *L. infantum promastigotes* showed increased activity against this parasitic disease when compared with the starting chloride forms and amphotericin B alone, highlighting [P_6,6,6,14_][AmB] as the most promising formulation. Possible synergism in the antiprotozoal activity was also evaluated for [P_6,6,6,14_][AmB], since it was proven to be the compound with the highest toxicity. This work reported a simple synthetic method, which can be applied to prepare other organic salts based on molecules containing fragile chemical groups, demonstrating the potential of these OSILs-AmB as possible agents against leishmaniasis.

## 1. Introduction

**Figure 1:**
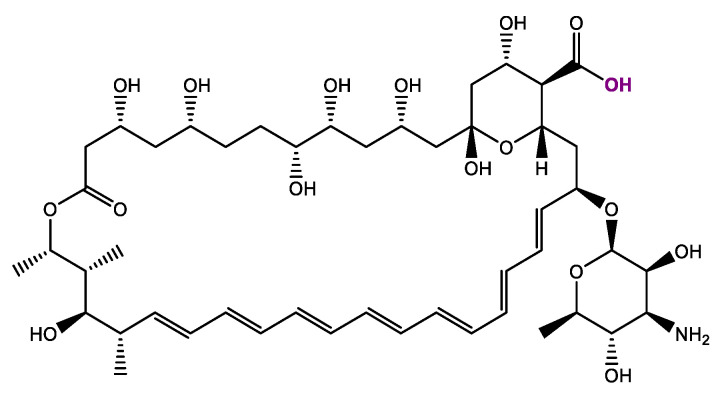
Structure of amphotericin B (AmBH) in its neutral form, evidencing the -OH group able to be deprotonated.

Amphotericin B (AmBH, Figure 1) is an antibiotic highly used against fungal infections [1,2] first introduced in 1958 and has been dominating the market over the years [3,4,5]. AmBH is a yellow solid product that can be found in both neutral and zwitterionic forms, possessing a polar and apolar side of the lactone ring, polyene chain and ionizable carboxyl and amine groups, which provide amphoteric properties to the molecule [6,7,8]. These physicochemical properties are the reason why AmBH is poorly soluble in aqueous media, as well as several organic solvents [6], leading, in some cases, to self-aggregation [8]. Since this pharmaceutical drug is insoluble in water, there are only two possible ways for drug administration: (i) through the addition of sodium desoxycholate to form a colloidal dispersion for intravenous infusion (Fungizone^®^) [1,9,10] and (ii) by single bilayer liposomal drug delivery system (Ambisome^®^) [1,11]. Nevertheless, both approaches present some drawbacks, such as nephrotoxicity [1,12] for the former and more frequent administrations are required for the latter, although this last approach allows a larger dose tolerance with reduced side effects.

AmBH is a low-soluble polyene antifungal, belonging to the mycosamine family, which includes nystatin, candicidin and rimodicin [13,14]. At an industrial scale, this drug is extracted from *Streptomyces nodosus* [6,8]. The total synthesis of AmBH was described in 1988 by Nicolaou et al. [15]. Nonetheless, biotechnological production is still a much cheaper and more efficient process. In addition, due to the intrinsic lack of stability associated with the presence of the labile hemiacetal group, chemically transforming AmBH in high yields (70% to quantitative) is quite challenging. This is only possible when using very reactive chemical reagents on amino and carboxylate groups or by first protecting these chemical groups to perform further modifications elsewhere [16,17,18,19,20,21]. Both approaches are experimentally laborious to execute and may lead to complications. Moreover, many reported cases do not even reach reaction yields above 60% [22], some being as low as 10% [23,24,25].

Organic salts and ionic liquids containing active pharmaceutical ingredients (OSILs-API) have emerged as a greener alternative to overcome problems in the pharmaceutical industry [26,27,28,29,30]. Moreover, it is also known that ionic liquids can provide a supplementary function to the active pharmaceutical ingredient (API) [31,32,33] and act as promising solvents, cosolvents and/or reagents to develop new APIs [33,34,35], being classified as the third generation of ionic liquids. In this approach, it is possible to take advantage of cation/anion fine-tuning to design compounds with improved physicochemical properties. Therefore, combining counterions with active pharmaceutical ingredients [36,37,38] can be relevant for solving solubility, membrane permeability and polymorphism, among other issues [39,40,41,42,43,44,45]. In this context, McCrary [46], Jameson [47] and their collaborators demonstrated that ionic liquid can be designed to modulate the hydro- and lipophilicity of AmBH, allowing this drug to surpass its solubility and aggregation problems.

Leishmaniasis is the generic term for protozoan parasitic diseases caused by several *Leishmania* spp., endemic in 98 countries and is of great public health concern, infecting approximately two million patients per year [48,49]. This disease is present in all areas favorable to the development of its vector, the sandfly. After biting their host, *Leishmania* promastigotes, the parasite’s flagellate stage, are transmitted and eventually mature into the amastigote stage in phagocytic cells, which are the target cells of this vector [48]. One of the most severe cases of this disease is visceral leishmaniasis (VL), being fatal without adequate treatment [50]. Since no vaccine was available for more than 70 years, the relatively inexpensive pentavalent antimonials were used as the first line of defense. For a long time, AmBH was considered a second-line treatment against VL, even though inherent problems related to its toxicity were always an issue. However, liposomal formulations emerged as a new approach to be used in a monotherapy regime, being also recommended by FDA and WHO [51,52]. AmBH has high affinity to ergosterol (membrane-bound sterol of fungi and protozoa) [53] and promotes the formation of small pores in the membrane of these microorganisms that modifies the permeability to cations, water and glucose [53,54] and disrupt the cell osmotic integrity, causing cell death [53,55]. Nevertheless, new methods to cure this disease have been proposed, including host-directed and combinational therapies, drug repurposing and nanotechnology. In addition, ionic liquids, other solvents and nanoparticles [18,19,56] have also played a role in what concerns the increase in drug solubilization and the improvement of formulations and delivery [17,57]. Thus, the search for organic salts or ionic liquids as a novel method for easy transformation of amphotericin B in high yields without additional protection/deprotection steps seems desirable to investigate.

Herein, we present a novel and innovative strategy to synthesize organic salts based on amphotericin B with different cations of ammonium, methylimidazolium, pyridinium and phosphonium type, namely [Aliquat][AmB], [Ch][AmB], [C_2_OHMIM][AmB], [C_3_OMIM][AmB], [C_16_Pyr][AmB] and [P_6,6,6,14_][AmB]. Moreover, the biological properties of these new compounds were evaluated against *Leishmania infantum*, aiming to understand their potential as a therapeutically antiprotozoal drug. In this context, to quantify the improvement in activity, the relative decrease in inhibitory concentration (RDIC) [27,58] was determined, being particularly relevant in the case of antibiotics [59,60]. This parameter can separate OSILs-API into two classes: (i) enhancer, when the activity increases up to one order of magnitude (~10-fold) and (ii) potentiator, if the activity increases two (~100-fold) or three (~1000-fold) orders of magnitude.

## 2. Materials and Methods

### 2.1. Synthesis and Chemical Characterization of OSILs Based on Amphotericin B

Chemical reagents and solvents were acquired from Aldrich, BDH, Solchemar and Valente & Ribeiro, being utilized without further purification, while the basic anion-exchange resin Amberlite IRA-400 (OH^−^) (ion-exchange capacity: 1.4 mEq mL^−1^) was supplied by Supelco. Regarding chemical characterization, ^1^H-NMR spectra were recorded on a Bruker AMX400 spectrometer at room temperature, using (CD_3_)_2_SO (from EurisoTop) as a deuterated solvent, and the chemical shifts were reported downfield in parts per million (ppm). FTIR spectra were measured on a Perkin Elmer 683. MALDI-TOF mass spectrometry was carried out on a Voyager-DE™ PRO Workstation model without matrix and with 3-HPA in both positive and negative ion modes.

**Scheme 1:**
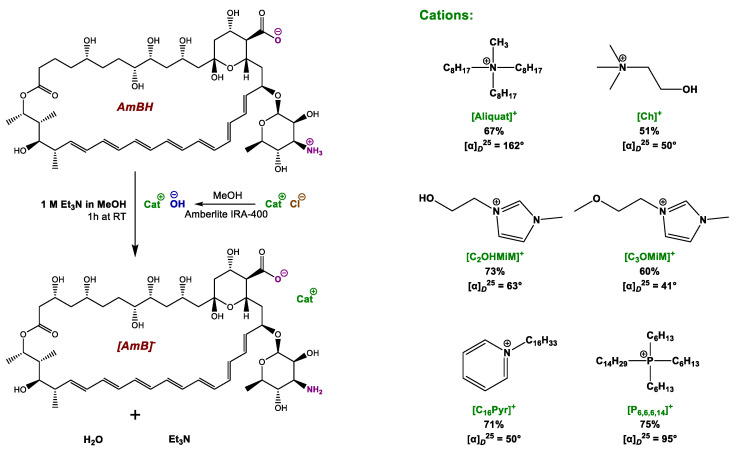
Synthesis of OSILs-AmB under water-reduced buffer neutralization conditions.

In this work, neutral/zwitterionic AmBH was deprotonated with Et_3_N and coupled as an anion to form OSILs-AmB with different ammonium, methylimidazolium, pyridinium and phosphonium organic cations. All compounds were prepared according to the following general procedure: (i) the selected organic cations were first transformed into hydroxides through Amberlite IRA-400 (OH^−^), an ionic exchange column, in methanol, as described in a previous publication [61]; (ii) the prepared hydroxides were then neutralized with the amphotericin B dissolved in 1 M dry triethylamine buffer methanolic solution and left to stir for 1 h at room temperature, whose method was adapted from Ferraz et al. [62]; (iii) aiming to purify the desired product, the solution was evaporated, redissolved in methanol, filtered through a calcium carbonate pad (3.0 g; 30.0 mmol) followed by evaporation and drying under vacuum. For more details, see Scheme 1 and Section 3.1.

**Synthesis of methyltrioctylammonium amphotericin B, [Aliquat][AmB]:**

Following the general procedure, tri-octylmethylammonium chloride (0.100 g; 0.246 mmol) was converted into hydroxide and, afterward, added to amphotericin B (0.251 g; 0.271 mmol) predissolved in 1 M dried triethylamine methanolic solution. The desired product was obtained as an orange solid (0.176 g; 67.1%). [α]*_D_*^25^: 162.0° ± 0.6 (c = 1 mg mL^−1^ in methanol); ^1^H-NMR (400.13 MHz, (CD_3_)_2_SO, Figure S1): δ = 6.47−6.05 (m, 16 H), 5.73 (bs, 1 H), 5.51−5.13 (m, 4 H), 4.79−3.36 (m, 24 H), 3.17 (t, 8 H, *J* = 7.5 Hz), 3.09−3.01 (m, 4 H), 2.92 (s, 3 H), 2.33−2.15 (m, 6 H) 1.86−1.71 (m, 4 H), 1.59 (bs, 8 H), 1.25 (m, 27 H), 1.15 (d, 3 H, *J* = 5.4 Hz), 1.11 (d, *J* = 5,4Hz, 3 H), 1.03 (d, 6 H, *J* = 5.5 Hz), 0.91 (d, 3 H, *J* = 6.8 Hz), 0.86 (bs, 12 H); FTIR (KBr, Figure S2): υ = 3441, 3014, 2925, 2854, 1716, 1690, 1638, 1617, 1579, 1565, 1458, 1402, 1383, 1324, 1271, 1181, 1126, 1110, 1070, 1037, 1011, 903, 854, 808, 719 cm^−1^; MALDI-TOF-MS analysis in positive ion mode: *m/z* calculated for C_25_H_54_N^+^ 368.4251, found 368.4251; and in negative ion mode: *m/z* calculated for C_47_H_72_NO_17_^−^ 922.4806, found [M-2H]^−^ 920.4777.

**Synthesis of choline amphotericin B, [Ch][AmB]:**

Following the general procedure, 2-hydroxy-ethyltrimethylammonium chloride (0.036 g; 0.254 mmol) was converted into hydroxide and, afterward, added to amphotericin B (0.249 g; 0.269 mmol) predissolved in 1 M dried triethylamine methanolic solution. The desired product was obtained as an orange solid (0.134 g; 51.1%). [α]*_D_*^25^: 50.0° ± 5.8 (c = 1 mg mL^−1^ in methanol); ^1^H-NMR (400.13 MHz, (CD_3_)_2_SO, Figure S3): δ = 6.47−5.97 (m, 14 H), 5.68 (bs, 1 H), 5.46−5.40 (m, 1 H), 5.35−5.32 (m, 1 H), 5.24−5.20 (m, 1 H), 4.98−4.74 (m, 1 H), 4.63 (bs, 1 H), 4.34 (bs, 1 H), 4.37−4.32 (m, 1 H), 4.26−4.24 (m, 1 H), 4.08−4.04 (m, 1 H), 3.84−3.83 (m, 6 H), 3.61−3.64 (m, 4 H), 3.60−3.38 (m, 10 H), 3.10 (s, 9 H), 2.33−2.27 (m, 3 H), 2.15 (d, 1 H, *J* = 5.8 Hz), 1.91−1.70 (m, 1 H), 1.65−1.31 (m, 10 H), 1.23 (s, 3 H), 1.14 (d, 3 H, *J* = 5.6 Hz), 1.10 (d, 3 H, *J* = 6.1 Hz), 1.03 (d, 3 H, *J* = 6.0 Hz), 0.91 (d, 3 H, *J =* 7.0 Hz), 0.83 (t, 3 H, *J* = 6.7 Hz) ppm; FTIR (KBr, Figure S4): υ = 3398, 3018, 2917, 2077, 1638, 1577, 1559, 1506, 1460, 1401, 1387, 1324, 1270, 1183, 1130, 1110, 1073, 1035, 982, 956, 851, 721 cm^−1^; MALDI-TOF-MS analysis in positive ion mode: *m/z* calculated for C_5_H_14_NO^+^ 104.1070, found 104.1080; and in negative ion mode: *m/z* calculated for C_47_H_72_NO_17_^−^ 922.4806, found [M-2H]^−^ 920.6717.

**Synthesis of 1-(2-hydroxyethyl)-3-methylimidazolium amphotericin B, [C_2_OHMIM][AmB]:**

Following the general procedure, 1-(2-hydroxyethyl)-3-methylimidazolium chloride (0.040 g; 0.226 mmol) was converted into hydroxide and, afterward, added to amphotericin B (0.251 g; 0.272 mmol) predissolved in 1 M dried triethylamine methanolic solution. The desired product was obtained as an orange solid (0.173 g; 72.8%). [α]*_D_*^25^: 63.0° ± 5.8 (c = 1 mg mL^−1^ in methanol); ^1^H-NMR (400.13 MHz, (CD_3_)_2_SO, Figure S5): δ = 9.10 (s, 1 H), 7.72 (s, 1 H), 7.69 (s, 1 H), 6.48−5.96 (m, 14 H), 5.69 (bs, 1 H), 5.53−5.40 (m, 1 H), 5.32 (bs, 1 H), 5.21−5.10 (m, 1 H), 4.86−4.70 (m, 2 H), 4.65−4.57 (m, 2 H), 4.44−4.33 (m, 2 H), 4.21 (t, 3 H, *J* = 4.9 Hz), 4.08−4.03 (m, 2 H), 3.86 (s, 3 H), 3.72 (t, 3 H, *J* = 4.9 Hz), 3.64−3.17 (m, 12 H), 3.13−2.84 (m, 5 H), 2.36−2.28 (m, 2 H), 2.16 (d, 1 H, *J* = 5.9 Hz), 1.86−1.23 (m, 16 H), 1.14 (d, 3 H, *J* = 5.9 Hz), 1.11 (d, 3 H, *J* = 6.1 Hz), 1.04−1.01 (m, 3 H), 0.91 (d, 3 H, *J =* 6.9 Hz) ppm; FTIR (KBr, Figure S6): υ = 3436, 2924, 2856, 1637, 1567, 1490, 1468, 1458, 1403, 1389, 1328, 1272, 1233, 1183, 1132, 1104, 1071, 1009, 978, 905, 857, 776, 721, 687 cm^−1^; MALDI-TOF-MS analysis in positive ion mode: *m/z* calculated for C_6_H_11_N_2_O^+^ 127.0866, found 127.0710; and in negative ion mode: *m/z* calculated for C_47_H_72_NO_17_^−^ 922.4806, found [M-2H]^−^ 920.4689.

**Synthesis of 1-(2-methoxyethyl)-3-methylimidazolium amphotericin B, [C_3_OMIM][AmB]:**

Following the general procedure, 1-(2-methoxyethyl)-3-methylimidazolium chloride (0.041 g; 0.252 mmol) was converted into hydroxide and, afterward, added to amphotericin B (0.251 g; 0.272 mmol) predissolved in 1 M dried triethylamine methanolic solution. The desired product was obtained as an orange solid (0.159 g; 59.5%). [α]*_D_*^25^: 41.0° ± 7.1 (c = 1 mg mL^−1^ in methanol); ^1^H-NMR (400.13 MHz, (CD_3_)_2_SO, Figure S7): δ = 9.11 (s, 1 H), 7.73 (s, 1 H), 7.70 (s, 1 H), 6.51−5.96 (m, 14 H), 5.71 (bs, 1 H), 5.53−5.40 (m, 2 H), 5.35 (bs, 1 H), 5.21−5.11 (m, 1 H), 4.85−4.63 (m, 5 H), 4.57−4.56 (s, 2 H), 4.36−4.34 (m, 4 H), 4.26−4.22 (m, 2 H), 4.19−4.14 (m, 2 H), 4.07−4.04 (m, 2 H), 3.58−3.36 (m, 12 H), 3.26 (s, 3 H), 2.33−2.25 (m, 2 H), 2.16 (d, 1 H, *J* = 5.6 Hz), 1.91−1.23 (m, 16 H), 1.15 (d, 3 H, *J* = 5.7 Hz), 1.11 (d, 3 H, *J* = 6.2 Hz), 1.04 (d, 3 H, *J* = 5.9 Hz), 0.91 (d, 3 H, *J =* 6.9 Hz) ppm; FTIR (KBr, Figure S8): υ = 3435, 2921, 2848, 1656, 1648, 1579, 1561, 1490, 1480, 1456, 1385, 1322, 1262, 1179, 1130, 1106, 1069, 1039, 1009, 901, 853, 776, 719, 685 cm^−1^; MALDI-TOF-MS analysis in positive ion mode: *m/z* calculated for C_6_H_11_N_2_O^+^ 127.0866, found 127.0710; and in negative ion mode: *m/z* calculated for C_47_H_72_NO_17_^−^ 922.4806, found [M-2H]^−^ 920.4689.

**Synthesis of cetylpyridinium amphotericin B, [C_16_Pyr][AmB]:**

Following the general procedure, cetylpyridinium chloride (0.088 g; 0.246 mmol) was converted into hydroxide and, afterward, added to amphotericin B (0.250 g; 0.271 mmol) predissolved in 1 M dried triethylamine methanolic solution. The desired product was obtained as an orange solid (0.215 g; 71.3%). [α]*_D_*^25^: 49.7° ± 5.8 (c = 0.2 mg mL^−1^ in methanol); ^1^H-NMR (400.13 MHz, (CD_3_)_2_SO, Figure S9): δ = 9.10 (d, 2 H, *J* = 5.6 Hz), 8.60 (t, 1 H, *J* = 7.6 Hz), 8.16 (t, 2 H, *J* = 6.7 Hz), 6.47−5.97 (m, 14 H), 5.65 (bs, 1 H), 5.51−5.40 (m, 1 H), 5.33−5.31 (m, 1 H), 5.21−5.20 (m, 1 H), 4.79−4.78 (m, 3 H), 4.59 (t, 3 H, *J* = 7.4 Hz), 4.34 (bs, 1 H), 4.24−4.23 (m, 2 H), 4.17−4.12 (m, 1 H), 4.07−4.05 (m, 1 H), 3.74−2.81 (m, 12 H), 2.41−2.25 (m, 3 H), 2.15 (d, 1 H, *J* = 5.7 Hz), 1.91−1.89 (m, 2 H), 1.82−1.37 (m, 16 H), 1.27−1.23 (m, 28 H), 1.14 (d, 3 H, *J* = 5.8 Hz), 1.11 (d, 3 H, *J* = 6.1 Hz), 1.03 (d, 3 H, *J* = 5.9 Hz), 0.90 (d, 3 H, *J =* 6.9 Hz), 0.85 (t, 3 H, *J* = 6.7 Hz) ppm; FTIR (KBr, Figure S10): υ = 3435, 3010, 2920, 2852, 1638, 1579, 1563, 1488, 1456, 1401, 1383, 1340, 1324, 1272, 1181, 1130, 1108, 1069, 1037, 1009, 982, 908, 887, 853, 772, 719, 683 cm^−1^; MALDI-TOF-MS analysis in positive ion mode: *m/z* calculated for C_21_H_38_N^+^ 304.2999, found 304.3117; and in negative ion mode: *m/z* calculated for C_47_H_72_NO_17_^−^ 922.4806, found [M-2H]^−^ 920.5183.

**Synthesis of trihexyltetradecylphosphonium amphotericin B, [P_6,6,6,14_][AmB]:**

Following the general procedure, trihexyltetradecylphosphonium chloride (0.127 g; 0.246 mmol) was converted into hydroxide and, afterward, added to amphotericin B (0.251 g; 0.270 mmol) predissolved in 1 M dried triethylamine methanolic solution. The desired product was obtained as an orange solid (0.195 g; 75.0%). [α]*_D_*^25^: 95.0° ± 3.8 (c = 1 mg mL^−1^ in methanol); ^1^H-NMR (400.13 MHz, (CD_3_)_2_SO, Figure S11): δ = 6.35−6.02 (m, 16 H), 5.71 (bs, 1 H), 5.53−5.21 (m, 6 H), 4.79−3.89 (m, 24 H), 2.12 (t, 12 H, *J* = 14.3 Hz), 1.47−1.37 (m, 24 H), 1.29−1.24 (m, 47 H), 1.13 (dd, 2 H, *J* = 16.5 and 5.9 Hz), 1.06−0.99 (m, 3 H), 0.87 (t, 12 H, *J =* 7.3 Hz) ppm; FTIR (KBr, Figure S12): υ = 3435, 2921, 2848, 1656, 1648, 1579, 1561, 1490, 1480, 1456, 1385, 1322, 1262, 1179, 1130, 1106, 1069, 1039, 1009, 901, 853, 776, 719, 685 cm^−1^; MALDI-TOF-MS analysis in positive ion mode: *m/z* calculated for C_32_H_68_P^+^ 483.51, found 483.4919; and analysis in negative ion mode: *m/z* calculated for C_47_H_72_NO_17_^−^ 922.4806, found [M-2H]^−^ 920.6147.

### 2.2. Biological Activity against Leishmania infantum

To evaluate the biological activity of amphotericin B organic salts, *Leishmania infantum* promastigotes were selected as a biologically relevant model to test the effectiveness of newly prepared compounds. In this context, a cloned line of virulent *L. infantum* (MHOM/MA/67/ITMAP-263) was maintained at 27 °C in RPMI 1640 medium supplemented with 10% FCS, 2 mM L-glutamine, 100 U mL^−1^ penicillin, 100 mg mL^−1^ streptomycin and 20 mM HEPES buffer, all acquired from Lonza. The promastigotes were subcultured each week with dilutions of 1 × 10^6^ parasites/mL. After 5 days, they became stationary, reaching a density of around 2 × 10^7^ parasites/mL. A maximum of 10 passages is recommended. After 10 passages, a new culture was started with parasites recovered from infected mice.

The efficacy of OSILs-AmB against the promastigote forms was evaluated using a modified resazurin-based assay. In this context, the compounds of interest were prepared by serial dilution in a 96-well plate with a final volume of 100 μL. Then, 100 μL of a 4 × 10^7^ parasites/mL parasite solution was added for a final density of 2 × 10^6^ parasites/mL in the well. After 72 h of incubation at 27 °C, 20 μL of a 0.5 mM resazurin solution was added and plates were incubated for further 4 h under the same conditions. Then, fluorescence was measured in excitation (540 nm) and emission (620 nm) wavelengths using a Synergy 2 Multi-Mode Reader (Biotek). For each individual assay, the Z factor was calculated through Z_f_ = 1 − [3 × (σ_p_ + σ_n_)/|μ_p_ − μ_n_|], where μ and σ are, respectively, the means and the standard deviations of both positive and negative controls and Z_f_ is the Z-Factor. In addition, the IC_50_ value was determined to evaluate the antiparasitic effect. A non-linear regression analysis was conducted on Prism 9 for Windows (Version 9.4.0), corresponding to the averages of the results obtained in at least two independent experiments. Lastly, the investigation of the potential synergism in the context of [P_6,6,6,14_][AmB] activity [63] was performed with Compusyn software 1.0 [63].

## 3. Results and Discussion

### 3.1. Synthesis and Chemical Characterization of OSILs Based on Amphotericin B

**Figure 2:**
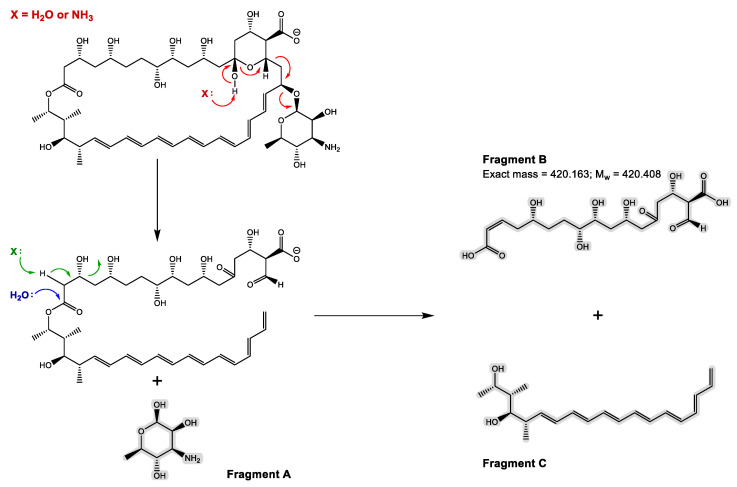
Grob fragmentation (red arrows), β-hydroxyl elimination (green arrows) and hydrolysis (blue arrow) of amphotericin B in aqueous ammonia solution.

Amphotericin B becomes unstable in the presence of light and oxygen, being also difficult to solubilize in most common organic solvents and at low pH [6,64]. Thus, in order to synthesize OSILs-AmB, a buffer neutralization method (1 M ammonium solution media) was first performed, aiming to stabilize the neutral/zwitterionic AmBH, a strategy previously implemented in the case of ampicillin [62] (also unstable, insoluble and in the zwitterionic form). However, the ammonia buffer solution could not protect amphotericin B from degradation and the resulting ^1^H-NMR spectra were unclear. Additionally, the desired [AmB]^−^ molecular peak (*m/z* 922 in negative mode) was absent in MALDI-TOF-MS spectra, as only one fragment was detected with *m/z* 420. One possible explanation is the occurrence of Grob fragmentation (Figure 2) starting from the labile hemiacetal group together with the elimination and hydrolysis of β-hydroxyester, originating Fragment B. For the case herein studied, the fragmentation of amphotericin B molecule might have started in the labile hemiacetal group promoted by the attack of an aqueous base with consecutive separation of the amino sugar (Fragment A), as depicted in Figure 2. Furthermore, elimination of the ester β-hydroxyl group is promoted by an aqueous base and followed by ester hydrolysis to form Fragment B. All three reactions are plausible in a basic aqueous solution. Therefore, as alternative, several other reaction conditions and solvents were tested, although degradation was always present and easily recognizable experimentally by precipitation of some crystalline substance from amphotericin B solutions (presumably, amino sugar—Fragment A).

Finally, after many unsuccessful attempts, solubilization of the neutral/zwitterionic AmBH was achieved with a methanolic solution of dry triethylamine. Since no sign of degradation was identified, the neutralization reaction was performed under these water-reduced buffer conditions (Scheme 1). In this context, amphotericin B was dissolved in 1 M dry triethylamine methanolic solution and, then, the solution containing the selected organic cation in its hydroxide form was added. The reaction mixture was left to stir for 1 h at room temperature, after which the solvent was evaporated and the resulting compound was purified by crystallization and subsequent filtration of the methanolic solution through a calcium carbonate pad. Lastly, the solvent was again evaporated and the compounds were isolated pure in moderate to high yields (51–75%, see Section 2.1). The prepared OSILs-AmB were characterized by ^1^H-NMR, as well as FTIR and MALDI-TOF analyses in positive and negative ion modes. The latter technique confirmed, on the positive and negative ion modes, the presence of the respective cation and the [M-H_2_]^−^ ion derived from the [AmB] anion [21]. Moreover, the specific optical rotation ([α]*_D_*^25^) of the prepared compounds was also measured and is depicted in Scheme 1. Interestingly, the results obtained followed the same trends observed in ionic liquids based on chiral amino acids. Carreira et al. [65] reported that the optical rotation magnitude of amino acid’s chiral precursors is higher than the ones detected for ionic derivatives. Herein, it is known that the [α]*_D_*^24^ of neat amphotericin B is 333° in acidic N,N-dimethylformamide conditions [66]. When coupled with the studied organic cations, the [α]*_D_*^25^ found were between 41° and 162°. In addition, even though both [Aliquat][AmB] and [Ch][AmB] are substituted tetralkylammonium derivatives, the magnitude of optical rotations is significantly different, being 162° for the first and 50° for the latter, which was also registered for analogues derivatives by Carreira et al. [66]. Furthermore, the [α]*_D_*^25^ values might be a useful tool for monitoring the decomposition of the prepared compounds due to the low stability of amphotericin B and its derivatives in solution, avoiding the use of more sophisticated techniques. It is worth noting that no degradation was detected during the experimental work reported.

### 3.2. Biological Activity against Leishmania infantum

Aiming to evaluate the biological activity against *Leishmania infantum* promastigotes of the prepared compounds, the half maximal inhibitory concentration (IC_50_) of both OSILs-AmB and the starting organic cations in their chloride form was determined. Moreover, the relative decrease in IC_50_ (RDIC) of each formulation containing the API was also calculated. The results collected are displayed in Table 1. Figures S13 and S14 comprise complementary information related to the antiparasitic activity.

Regarding the IC_50_ obtained by this biological assay, [P_6,6,6,14_][AmB] (61.4 nM) exhibited the lowest value, indicating the highest growth inhibition capacity when compared to the original AmBH (86.6 nM). This implies that is required less quantity to achieve the same biological outcome and, therefore, this formulation is the most promising one. The remaining OSILs-AmB were classified according to the decrease in antiparasitic activity: [Aliquat][AmB] > [C_3_OMIM][AmB] > [C_16_Pyr][AmB] > [Ch][AmB] > [C_2_OHMIM][AmB]. For the organic cations in their chloride form, only [P_6,6,6,14_][Cl], [Aliquat][Cl] and [C_16_Pyr][Cl] present anti-*Leishmania* activity in the tested concentrations range. Nonetheless, the obtained values are not statistically relevant since they are much higher than the IC_50_ value of AmBH. All compounds were tested in order to demonstrate if the starting materials exhibit any activity against this protozoan disease, allowing the conclusion that the improvements concerning the biological activity of OSILs-AmB are associated with the incorporation of this pharmaceutical drug. 

In order to fully understand the impact of each formulation in the anti-*Leishmania* activity when compared with API, the relative decreases in inhibitory concentrations (RDIC) were calculated. Higher RDIC values are desirable as they indicate increased activity of the OSILs-AmB in respect to the starting agent. These values, displayed in Table 1, highlight that only [P_6,6,6,14_][AmB] is more active against *L. infantum* promastigotes than AmBH alone. Moreover, the other OSILs-AmB exhibited values below 1, with the exception of [Aliquat][AmB] and [C_3_OMIM][AmB], which registered very similar biological activity to AmBH.

In general, the improvements detected in the antileishmanial activity promoted by the formulations herein studied are similar to the ones reported for homologues compounds in antifungal assays [61]. In both cases, fungi and protozoa, the drug’s mechanism of action is the same and translates into the interaction with ergosterol, found in cell membranes, and the consequent membrane disruption. Moreover, the contribution of some hydrophobic organic cations may provide some enhancements in bioactivity, although individual effects are not necessarily identical (e.g., the RDIC obtained here for [C_16_Pyr][AmB] is 0.84, while the same formulation exhibit a positive effect against fungi). On the contrary, for polar cations, as choline, an opposite phenomenon can occur, probably, due to ion trapping of AmBH in solution, as previously reported for tests conducted on fungi [61].

Lastly, as mentioned before, [P_6,6,6,14_][AmB] is the most promising formulation, revealing higher antiparasitic activity. Therefore, the possible synergism in the antiprotozoal activity was evaluated, since other combinatorial therapies with AmBH have already demonstrated significant synergistic effects responsible for the improvement in their therapeutic index [61]. Considering the relevant counterion activity, we evaluated the possibility of synergy in the antiprotozoal activity. This concept is well established and describes the enhancing effect associated with individual distinct chemical entities that, when combined, present an effect that is greater than the predicted sum of the individual effects. For example, the activity of two agents seems significantly different when compared to the prevalent antagonistic interaction of bactericidal and bacteriostatic antibiotics when administered jointly. Upon evaluation of synergism, using the median effect analysis, we observed that the antiparasitic potency of [P_6,6,6,14_]^+^ is sufficient to partially overlap the [AmB]^−^ potency, leading to a small incremental potency (Figures S13 and S14). The isobologram plot of [P_6,6,6,14_][AmB] (Figure S15) suggests that the activity of [P_6,6,6,14_]^+^ was nearly additive to the activity of [AmB]^−^. Overall, these results show that IL combination could be a good alternative to enhance amphotericin B as an anti-Leishmanial. Further studies to find new counterions with antiparasitic potential and possible synergic effects with [AmB]^−^ are desired, due to the possibility of enhancing the AmBH antiparasitic activity, while reducing simultaneously the risk of treatment failure associated to the generation of resistant parasites. 

## 4. Conclusions

This work emphasizes the use of a simple buffer neutralization method to synthesize organic salts and ionic liquids based on complex antimicrobial agents, such as amphotericin B or other molecules containing fragile chemical groups. The crucial step to successfully prepare the desired products is dissolving the API in 1 M dry triethylamine buffer methanolic solution, which impaired the occurrence of Grob fragmentation and the consequent degradation of AmBH. Furthermore, this innovative synthetic procedure allowed OSILs-AmB to be obtained in high yields and purity levels through an easier and faster process. The resulting compounds were characterized by NMR, FTIR and MALDI-TOF-MS, which confirmed the desired chemical structure as well as the suitable proportion between cation and anion. The specific optical rotation ([α]*_D_*^25^) was also measured, revealing values that are in agreement with the ones already reported for other organic salts combined with chiral molecules.

Since AmBH has proven itself effective against leishmaniasis, bioactivity studies were conducted to evaluate the therapeutic potential of the prepared formulations. In this context, IC_50_ and RDIC assays were performed, which suggested that only [P_6,6,6,14_][AmB] has higher activity than AmBH alone. The potential synergism in the antiprotozoal activity was also evaluated, showing the enhancing effect of the starting chloride organic salt form, leading to an increase in the activity of this prepared OSIL-AmB. This is quite a promising result that prompts the way for the development of new bioactive formulations with AmBH.

## Figures and Tables

**Table 1:** IC_50_ of OSILs-AmB and organic cations in their chloride form, as well as the RDIC obtained for formulations containing the API.

Compound	IC_50_ ^1^ (nM)	RDIC ^2^
AmBH	86.6 [80.67 to 92.96]	-
[Aliquat][Cl]	299.7 [190.1 to 472.3]	-
[Ch][Cl]	N.A.	-
[C_2_OHMIM][Cl]	N.A.	-
[C_3_OMIM][Cl]	N.A.	-
[C_16_Pyr][Cl]	482.8 [356.1 to 654.5]	-
[P_6,6,6,14_][Cl]	204.4 [183.2 to 228.2]	-
[Aliquat][AmB]	80.38 [73.39 to 88.03]	1.08
[Ch][AmB]	109.6 [95.64 to 125.6]	0.79
[C_2_OHMIM][AmB]	119.5 [99.10 to 144.0]	0.72
[C_3_OMIM][AmB]	88.26 [78.60 to 99.11]	0.98
[C_16_Pyr][AmB]	103.7 [90.53 to 118.7]	0.84
[P_6,6,6,14_][AmB]	61.4 [53.68 to 70.21]	1.41

^1^ The IC_50_ 95% confidence intervals are provided between brackets. ^2^ RDIC is the relative decrease in IC_50_ (RDIC = IC_50___AmB_/IC_50_OSILs-AmB_). N.A.—not active in the concentration range tested.

## Data Availability

Data are available in a publicly accessible repository.

## Supplementary Materials

The following supporting information can be downloaded at: https://www.mdpi.com/article/10.3390/antibiotics11121841/s1, Figures S1–S12: ^1^H-NMR and FTIR spectra; Figure S13: Antiparasitic activity of AmBH and each OSIL-AmB; Figure S14: Graphical representation depicting the average IC_50_ with 95% confidence interval; Figure S15: Isobologram and combination indexes of [P_6,6,6,14_][AmB] and their individual moieties.
