# Supplementary material for: Synthesis and Biological Evaluation of Amphotericin B Formulations Based on Organic Salts and Ionic Liquids against Leishmania infantum
Source: Antibiotics (Basel). 2022 Dec 19;11(12):1841. doi: 10.3390/antibiotics11121841 (PMC9774544; doi:10.3390/antibiotics11121841)
Supplement: Supplementary file 1 [file antibiotics-11-01841-s001.zip › antibiotics-2039284-supplementary.pdf]

Electronic Supplementary Information

Synthesis and Biological Evaluation of Amphotericin B based on Organic Salts and Ionic Liquids against *Leishmania Infantum*

Ricardo Ferraz [1,2,*,†], Nuno Santarém [3,†], Andreia F. M. Santos [4], Manuel L. Jacinto [4], Anabela Cordeiro-da-Silva [3,5], Cristina Prudêncio [1,6], João Paulo Noronha [4], Luis C. Branco [4,*] and Željko Petrovski [4,*]

[1] Chemical and Biomolecular Sciences, School of Health, Polytechnic Institute of Porto, 4200-072 Porto, Portugal
[2] LAQV-REQUIMTE, Department of Chemistry and Biochemistry, Faculty of Sciences, University of Porto, 4169-007 Porto, Portugal
[3] Institute for Research and Innovation in Health (i3S), University of Porto, 4200-135 Porto, Portugal
[4] LAQV-REQUIMTE, Department of Chemistry, NOVA School of Science and Technology, NOVA University of Lisbon, 2829-516 Caparica, Portugal
[5] Laboratory of Microbiology, Department of Biological Sciences, Faculty of Pharmacy, University of Porto, 4050-313 Porto, Portugal
[6] Center for Translational Health and Medical Biotechnology Research, Polytechnic Institute of Porto, 4200-465 Porto, Portugal
* Correspondence: ricardoferraz@eu.ipp.pt (R.F.); l.branco@fct.unl.pt (L.C.B.); z.petrovski@fct.unl.pt (Ž.P.)
† These authors contributed equally to this work.

Synthesis of OSILs-AmB

In order to minimize the impact from Grob fragmentation and ester hydrolysis, the following OSILs-API based on amphotericin B were prepared using dried solvents (water-free conditions at the beginning of the reaction):

1. [Aliquat][AmB]
2. [Ch][AmB]
3. [C_2OHMIM][AmB]
4. [C_3OMIM][AmB]
5. [C_{16}Pyr][AmB]
6. [$P_{6,6,6,14}$][AmB]

1. [Aliquat][AmB]

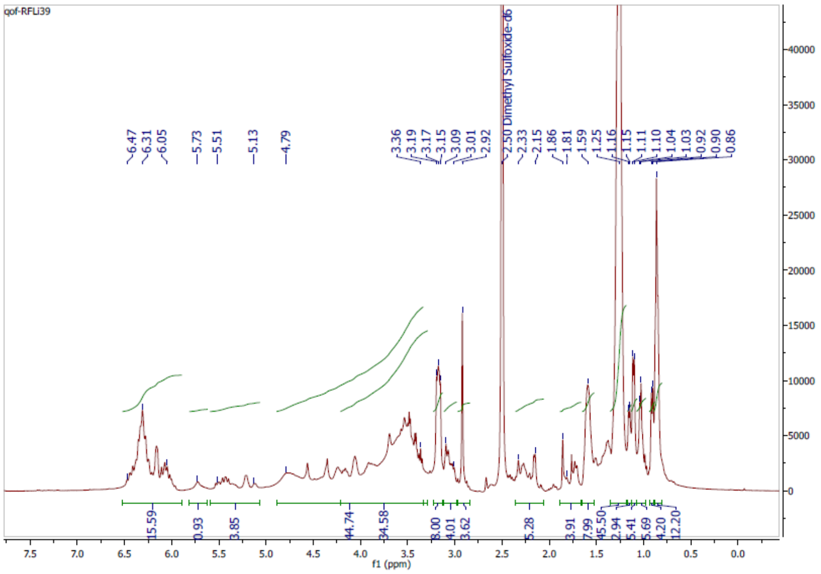

Figure S1: [Aliquat][AmB] ^{1}H-NMR spectrum in $(CD_3)_2SO$

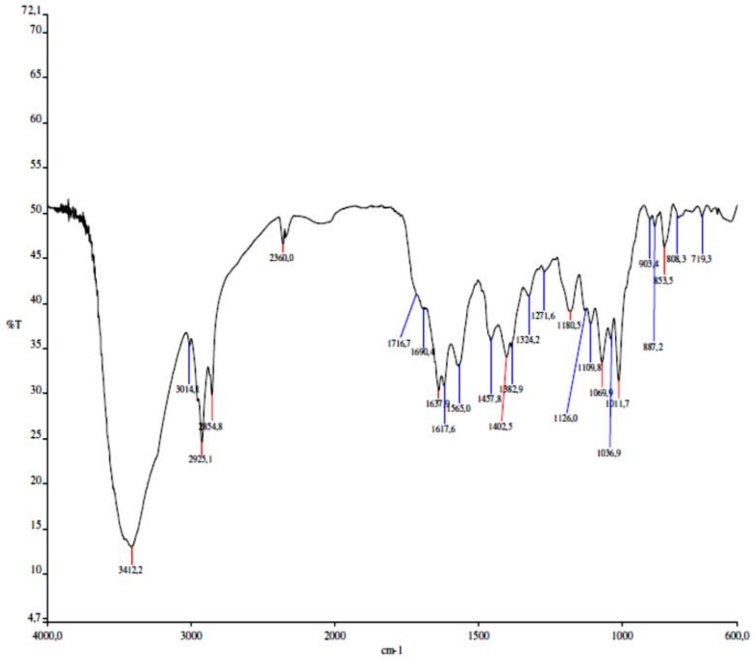

Figure S2: [Aliquat][AmB] FTIR spectrum in KBr

2. [Ch][AmB]

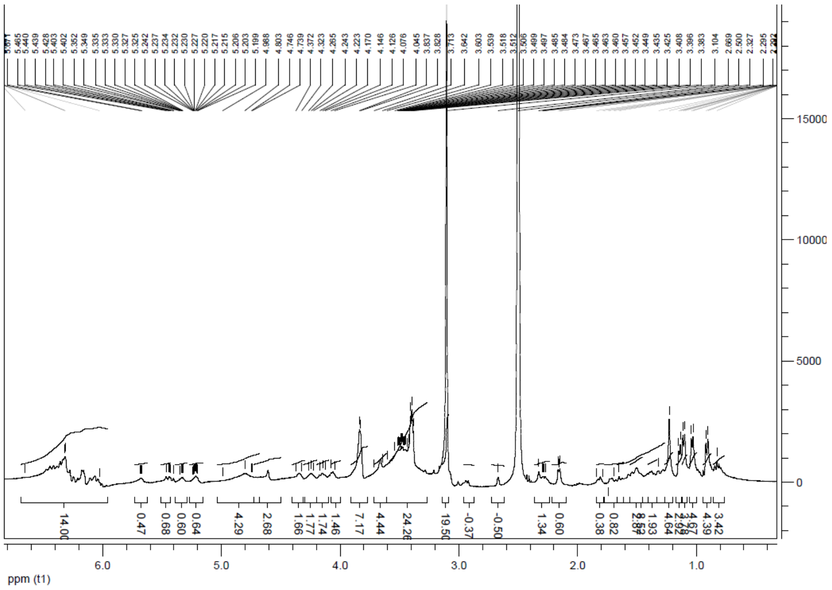

Figure S3: [Ch][AmB] ^{1}H-NMR spectrum in $(CD_3)_2SO$

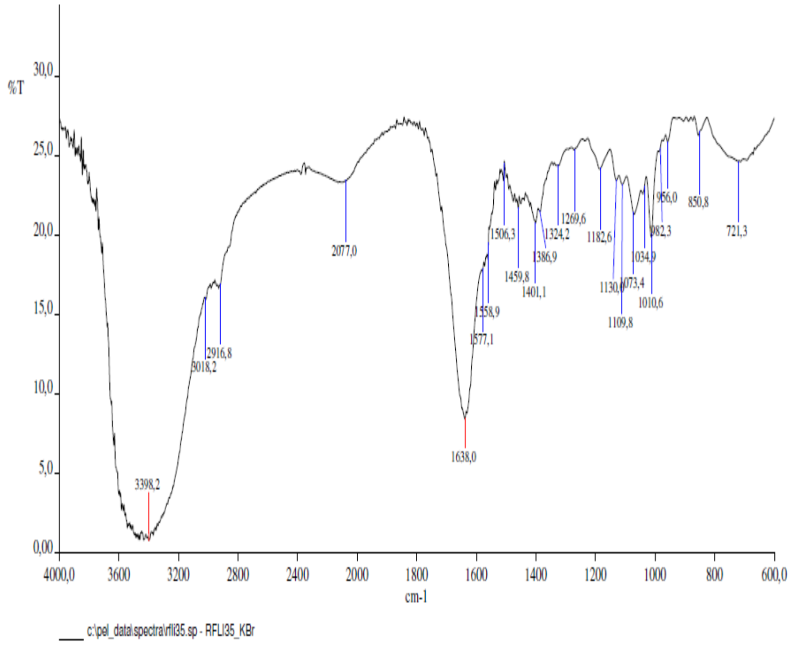

Figure S4: [Ch][AmB] FTIR spectrum in KBr

3. [C_2OHMIM][AmB]

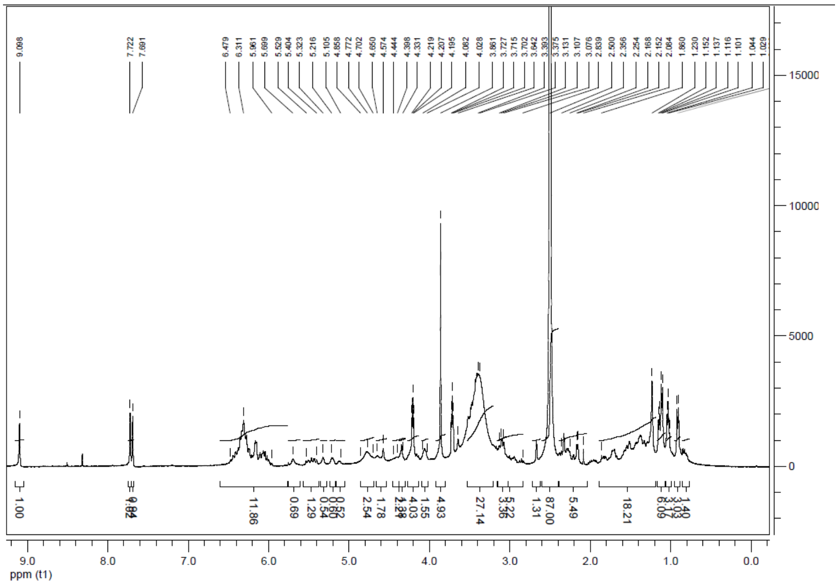

Figure S5: [C_2OHMIM][AmB] ^{1}H-NMR spectrum in $(CD_3)_2SO$

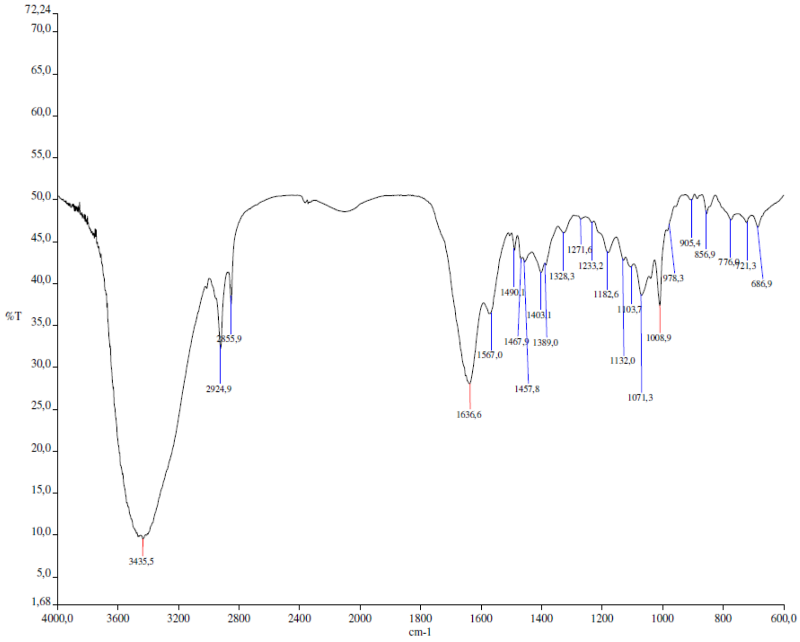

Figure S6: [C_2OHMIM][AmB] FTIR spectrum in KBr

4. [C_3OMIM][AmB]

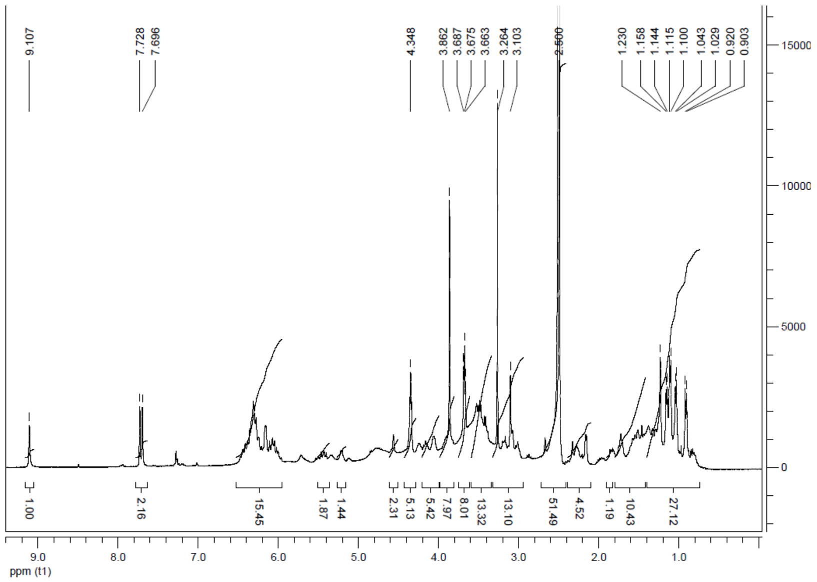

Figure S7: [C_3OMIM][AmB] ^{1}H-NMR spectrum in $(CD_3)_2SO$

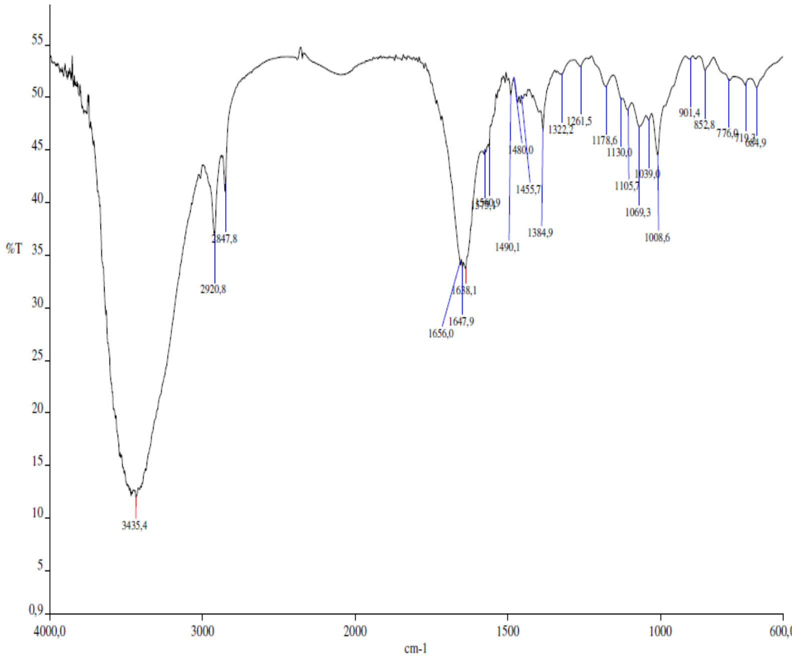

Figure S8: [C_3OMIM][AmB] FTIR spectrum in KBr

5. [C$_{16}$Pyr][AmB]

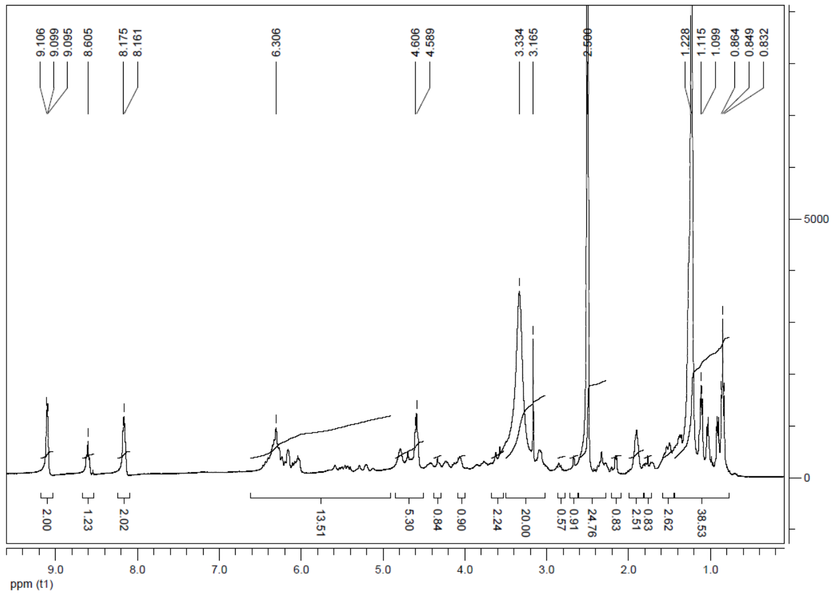

Figure S9: [C$_{16}$Pyr][AmB] ^{1}H-NMR spectrum in $(CD_3)_2SO$

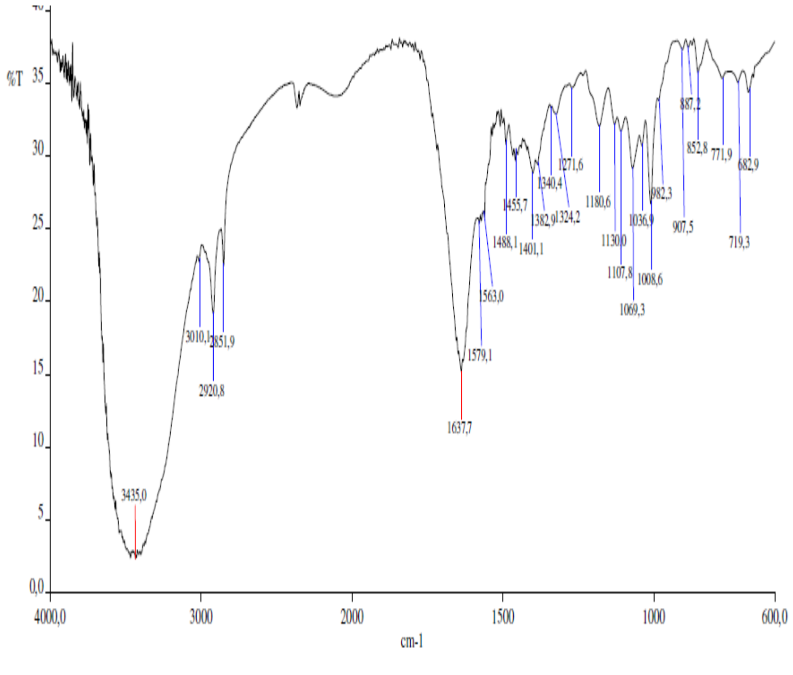

Figure S10: [C$_{16}$Pyr][AmB] FTIR spectrum in KBr

6. [$P_{6,6,6,14}$][AmB]

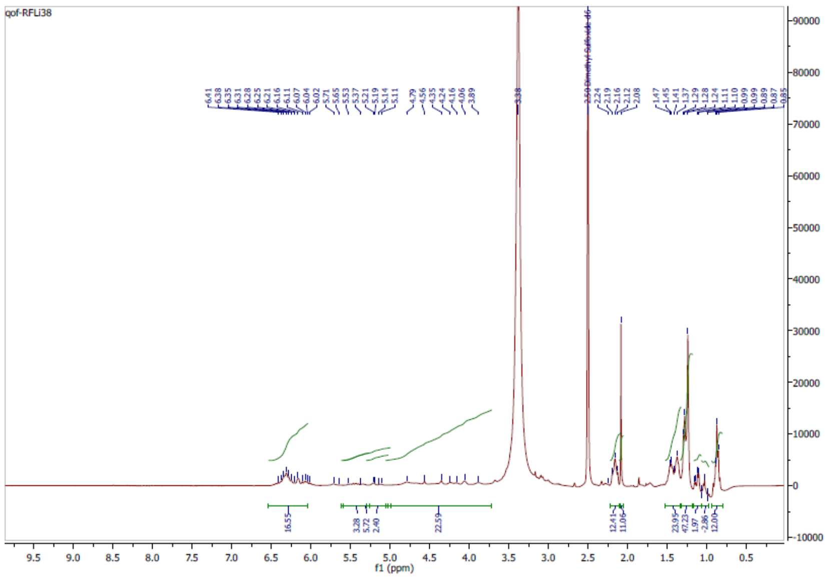

Figure S11: [$P_{6,6,6,14}$][AmB] ^{1}H-NMR spectrum in $(CD_3)_2SO$

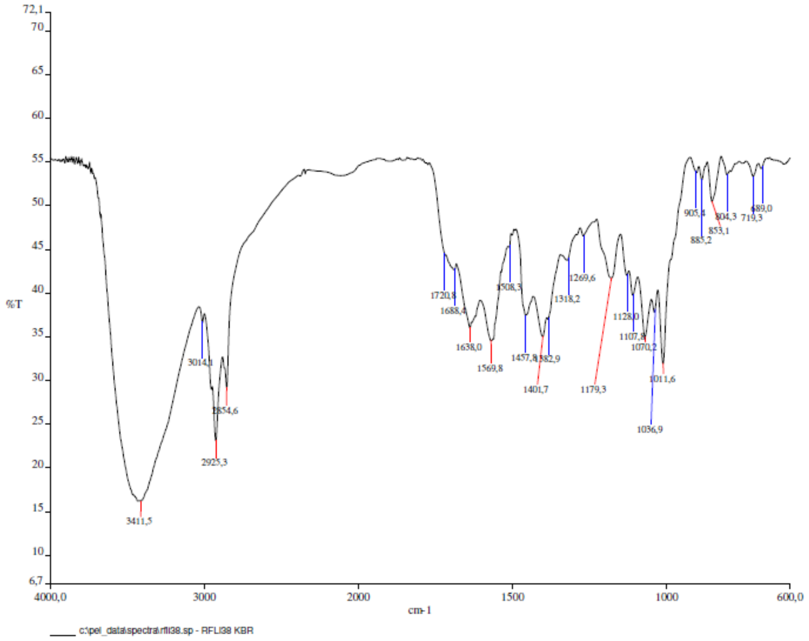

Figure S12: [$P_{6,6,6,14}$][AmB] FTIR spectrum in KBr

Biological Studies of OSILs-AmB

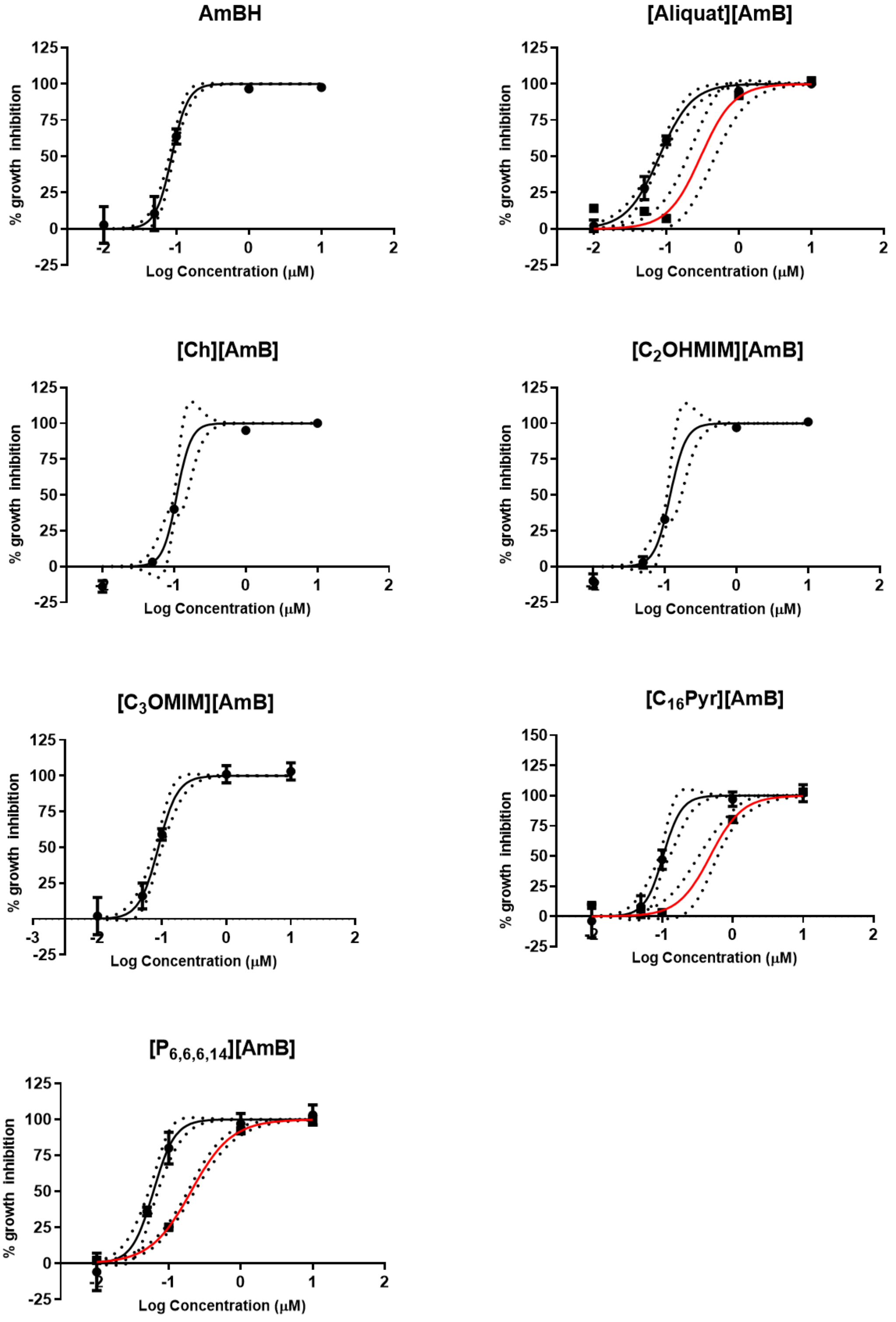

Figure S13: Antiparasitic activity of AmBH and each OSIL-AmB. Each point denotes the average growth inhibition and standard deviation for a defined concentration of AmphBH and OSILs-AmphB (•) or the respective organic cation in their chloride forms (▪). The solid black line and the solid red line represent the non-linear regression associated with AmBH and OSILs-AmB or the respective organic cation in their chloride forms, respectively. The dashed lines are associated with the non-linear regression, showing 95% confidence bands

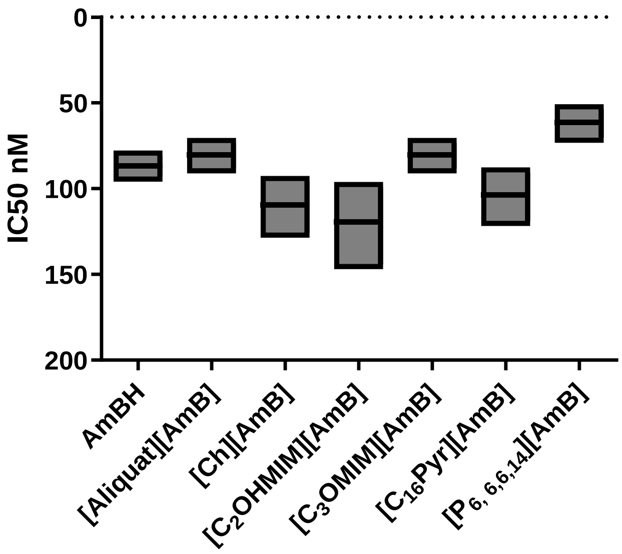

Figure S14: Graphical representation depicting the average IC_{50} with 95% confidence interval for AmBH and the prepared OSILs-AmB.

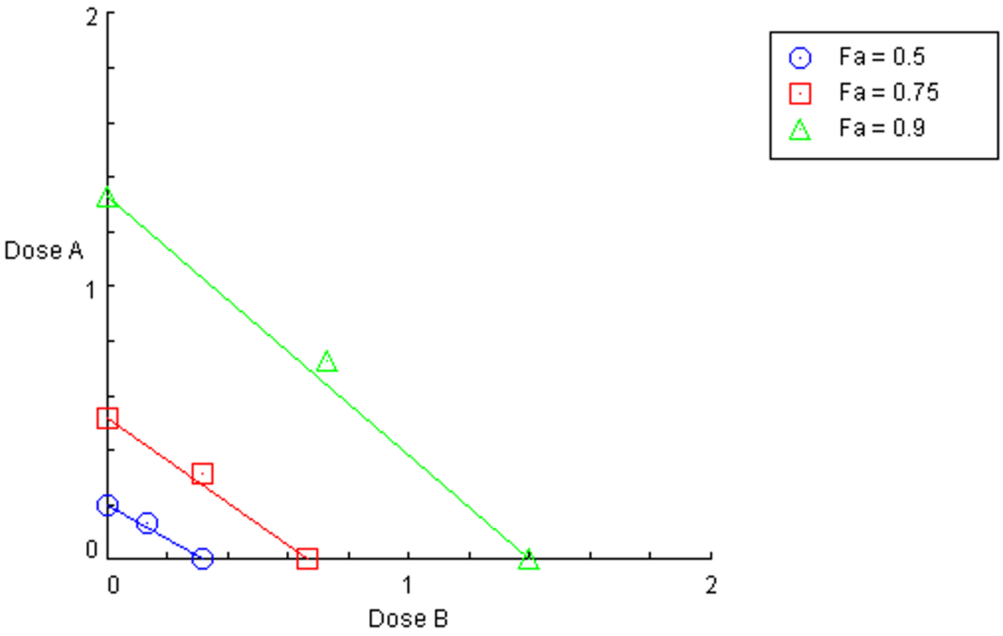

Figure S15: Isobologram and combination indexes (CIs) to compare the dose-effect of $[P_{6,6,6,14}][AmB]$ with their individual moieties: $[AmB]^-$ (compound A) and $[P_{6,6,6,14}]^+$ (compound B). The red, blue and green lines connect the predicted values of EC_{50} (blue), EC_{75} (red) and EC_{90} (green) for $[P_{6,6,6,14}]^+$ and $[AmB]^-$. The predicted CIs, calculated for EC_{50}, EC_{75} and EC_{90} values of the ionic liquid $[P_{6,6,6,14}][AmB]$, are 1.10, 1.08 and 1.06, respectively.
